# Formulation Screening and Characterization of PLGA-Based Injectable In Situ Gel Loaded with Progesterone

**DOI:** 10.3390/ph19091347

**Published:** 2026-08-26

**Authors:** Zhihan Zhu, Yu Liu, Linglin Feng

**Affiliations:** 1 NHC Key Lab of Reproduction Regulation, Shanghai Engineering Research Center of Reproductive Health Drug and Devices, Shanghai Institute for Biomedical and Pharmaceutical Technologies, Shanghai 200237, China; zhihanz5025@126.com; 2 Department of Pharmaceutics, School of Pharmacy, Fudan University, Shanghai 201203, China; liuyu@fudan.edu.cn; 3 State Key Laboratory of Advanced Drug Formulations for Overcoming Delivery Barriers, Shanghai 201203, China

**Keywords:** progesterone, sustained-release, injectable in situ gel, in vitro release, biocompatibility

## Abstract

**Objective:** Conventional progesterone (P4) formulations suffer from low bioavailability, severe local irritation, and poor patient adherence due to P4’s poor aqueous solubility. This work aimed to develop and screen a biodegradable PLGA/NMP (Poly(lactic-co-glycolic acid)/N-methyl-2-pyrrolidone) injectable in situ gel for sustained P4 delivery to overcome these clinical limitations. **Significance:** Commercial oral, vaginal, and oil-based intramuscular P4 preparations cannot maintain stable long-term drug exposure, and they cause injection-site pain/inflammation. The screened in situ depot system reduces administration frequency and local tissue irritation, supporting convenient luteal phase support and pregnancy maintenance. **Methods:** Nine formulations with variable P4 loading (10–50% w/w) and PLGA concentration (15–55% w/w) were fabricated. Formulations were screened via three core endpoints: injectability (injection force and discharge rate), in vitro sustained release in 10% Hydroxypropyl-β-cyclodextrin (HP-β-CD)-Phosphate-buffered saline (PBS) sink medium, and 7-day subcutaneous histocompatibility in rats. high-performance liquid chromatography (HPLC) was validated for progesterone quantification; Hematoxylin and eosin (H&E) staining assessed local inflammatory responses. **Results:** Formulations with progesterone ≤ 30% w/w and PLGA ≤ 35% w/w exhibited acceptable injectability (injection force < 50 N; discharge rate > 79%). Higher PLGA concentrations suppressed initial burst release (16.74% at 8 h for 35% PLGA vs. 29.8% for 20% PLGA). All formulations formed stable ellipsoidal subcutaneous depots and completed progesterone release within 4 days. Histopathology revealed only mild local inflammation (histological score = 1) without severe necrosis, superior to highly irritating oil injections in formulation control groups. **Conclusions:** The screened PLGA-based progesterone in situ gel resolves critical drawbacks of traditional progesterone dosage forms. This low-irritation, sustained-release injectable platform provides a scalable industrial formulation candidate for long-acting hormone therapy.

## 1. Introduction

Preterm birth is defined as delivery before 37 weeks of gestational age and is categorized into three subgroups: extremely preterm (<28 weeks), very preterm (28–32 weeks), and moderate to late preterm (32–37 weeks). According to UNICEF data, 152 million infants were born preterm in the past decade, making preterm birth the leading cause of neonatal death and the second leading cause of death among children under five years of age [1,2]. The survived preterm babies are more likely to have additional serious illness or even death during their neonatal period, and would have an increasing risk of lifelong disability and may lead to poor life quality [3]. What is worse, the trend of preterm birth has been increasing during the last two decades in most countries [4].

How to prevent preterm birth and make effective treatment to reduce maternal and neonatal complications becomes one of the biggest challenges in obstetrics [5,6]. Progesterone is known as a key steroid hormone, and it plays a very essential role during the whole process of pregnancy, with another name of pro-gestational steroid. The molecular formula of progesterone is C_21_H_30_O_2_, and the molecular weight is 314.47 g/mol. Progesterone exhibits thermal stability up to 174 °C, with a single decomposition process occurring between 174 and 339 °C (DTG peak at 312 °C), and a melting point at 131 °C (ΔH = 85.56 J·g^−1^) [7]. Its aqueous solubility is extremely low, with a reported value of 0.007 mg/mL at 25 °C, consistent with its high lipophilicity (logP = 3.87) [8]. The Biopharmaceutical Classification System (BCS) classification of progesterone remains a subject of debate within the literature. Progesterone is commonly designated as BCS Class II (low solubility and high permeability) due to its high lipophilicity and favorable in vivo absorption [7]. However, alternative studies, often based on in vitro model systems, have reported lower permeability estimates, leading to its classification as a BCS Class IV compound (low solubility and low permeability) [9]. As described in the FDA-approved product labels for progesterone injectable solutions, progesterone is practically insoluble in water but is sparingly soluble in vegetable oils, including sesame oil [10]. Therefore, the progesterone in oil preparations is advantaged to ensure higher drug concentration, which enhances the efficacy of maintaining pregnancy. The parenteral administration could ensure measurable and constant circulation plasma concentration of progesterone [11]. However, when administered intramuscularly, significant pain and discomfort follow, including local soreness, occasionally sterile abscess formation, severe inflammatory, and allergic reactions due to the local tissue disruption caused by the vehicle oil [12]. The convenience of oral administration is indisputable, and it is considered to be the best way for patients to be compliant. However, oral administration of progesterone is not only limited to the extensive first-pass metabolism in liver, but also to significant variability of individual plasma concentration [13,14]. The convenience of oral administration is indisputable and is considered the best way to ensure patient compliance. However, oral administration of progesterone is limited not only by extensive first-pass metabolism but also by significant inter-individual variability in plasma concentrations. This variability arises from multiple factors, including (1) food effects on gastrointestinal absorption, (2) extensive and variable first-pass hepatic metabolism, and (3) the significant contribution of intestinal metabolism, which can differ considerably between individuals and species [15,16]. Compared with oral administration, vaginal administration results in a higher peak plasma concentration (Cmax) but a more sustained and constant plasma level. For example, vaginal progesterone gel (Crinone^®^ 8%, 90 mg) achieves a Cmax of 10.51 ± 0.46 ng/mL with a Tmax of 7.67 ± 3.67 h, whereas a 100 mg oral capsule produces a Cmax of only 2.20 ± 3.06 ng/mL but with a Tmax of 1.00 ± 0.41 h. The vaginal route also exhibits less inter-individual variability in absorption, approximately 40-fold less for Cmax, and significantly higher dose-normalized AUC (1.48 ± 0.16 vs. 0.035 ± 0.052 ng·h/mL per mg). The higher Cmax observed with vaginal administration at comparable doses is explained by the “uterine first-pass effect,” where the drug is preferentially delivered to the uterus via direct diffusion, resulting in higher systemic concentrations while maintaining high local tissue levels [17]. Vaginal administration is highly preferable in women health application; it is effective in the maintaining of pregnancy through inducing the secretory transformation of the endometrium and preventing cervix prematurely shortening [18,19]. However, vaginal application is associated with several drawbacks related to the dosage form or delivery device. Gel formulations (e.g., Crinone^®^ 8%) are often reported to cause leakage and a bothersome buildup of the gel matrix, which can lead to local irritation and itching; vaginal tablets (e.g., Endometrin^®^) are more frequently linked to increased vaginal discharge; and intravaginal rings (IVRs) present challenges such as the risk of unintended expulsion and foreign body sensation [13,20]. It is urgent to develop minimally invasive, long-acting injectable progesterone delivery systems with mild local irritation and simplified dosing regimens to cut administration frequency and stabilizing systemic drug exposure.

Polymer-based in situ forming depots stand out as a promising class of sustained-release injectables, which are promising advancement in the delivery of poorly water-soluble drugs, such as progesterone and BCS II/IV drugs. These systems exist as low-viscosity solutions at room temperature, which facilitates manufacturing operations and can be easily injected by normal medical needle (23-gauge needles with inner diameter of about 0.337 mm), and then undergo a rapid sol-to-gel phase transition upon exposure to aqueous physiological fluids in the body [21,22]. This transition is mainly driven by solvent exchange, in which a water-miscible organic solvent diffuses into the surrounding tissue fluid, prompting the dissolved biodegradable polymer to precipitate and form a solid-like, localized drug depot [22]. By establishing a subcutaneous or intramuscular drug reservoir, such in situ gel systems enable controlled and prolonged drug release over days to weeks, bypass hepatic first-pass metabolism to improve systemic bioavailability, and significantly reduce dosing frequency [23].

Poly(lactic-co-glycolic acid), known as PLGA, is a well-studied biodegradable polymer that is widely used to fabricate drug-loaded nanoparticles capable of realizing sustained drug release from days to weeks, and even months [24]. Benefiting from favorable biocompatible performance, complete biodegradation capacity, and satisfactory toxicological profiles, this polymer material has obtained official marketing approvals issued by both the U.S. Food and Drug Administration (FDA) and European Medicines Agency (EMA) [25,26]. NMP (N-methyl-2-pyrrolidone) is a well-recognized pharmaceutical excipient that is critical to the performance of injectable in situ gel systems. Following subcutaneous administration, its rapid diffusion into the aqueous physiological environment (i.e., interstitial fluid at the injection site) triggers instantaneous PLGA precipitation, leading to the formation of a stable in situ gel depot [26]. NMP-triggered PLGA in situ gel avoids extra crosslinking operation; possesses scalable formulation manufacturing advantages; and has been widely exploited for poorly water-soluble steroid, polypeptide, and small-molecule sustained release [21].

In this study, we developed a series of biodegradable, sustained-release injectable progesterone-loaded PLGA/NMP injectable in situ gels with varying polymer concentrations and drug loading. Both progesterone loading and polymer concentration are expected to critically influence formulations in injectability, burst release, and local tissue compatibility. A systematic screening approach was therefore designed to identify the progesterone sustained-release injectable. The formulation was developed and screened via evaluation of key parameters: (i) to evaluate injectability, evaluated by measuring injection force and discharge rate through clinically relevant needle gauges to ensure patient-friendly administration; (ii) to characterize in vitro drug release behavior and identify key formulation parameters controlling burst release and overall release kinetics; and (iii) to assess local biocompatibility in comparison with the commercial oil-based progesterone injection in a rat model.

This study was aimed to provide a systematic screening approach for identifying formulation constraints and composition–performance relationships in PLGA-based in situ gel systems, which may inform the development of other lipophilic drug depots. Second, it offers a practical and scalable formulation platform that has the potential to reduce dosing frequency while maintaining acceptable local tolerability, addressing a potential clinical preparation in progesterone therapy.

## 2. Results

### 2.1. Equilibrium Solubility of Progesterone

The experimental results showed that the equilibrium solubility of progesterone increased with the increasing of HP-β-CD concentration in the PBS solution. When the HP-β-CD concentration was 6%, the solubility of progesterone was 4.71 ± 0.13 mg/mL; as the concentration increased to 10%, the solubility reached 7.61 ± 0.15 mg/mL. A further increase in progesterone concentration in 15% HP-β-CD-PBS solution was observed to 11.0 ± 0.44 mg/mL. While the concentration of progesterone in NMP was 187.91 ± 0.03 mg/mL. Considering factors such as solubility enhancement efficiency, economic cost, and potential influence on subsequent in vitro release behavior, 10% HP-β-CD/PBS was finally selected as the in vitro release medium for subsequent experiments.

Sink condition validation confirmed that the dissolved progesterone concentration in the 100 mL release medium remained well below the equilibrium solubility (7.6 mg/mL) throughout the release study, meeting the FDA’s sink condition requirement for in vitro drug release studies.

### 2.2. Stability of Progesterone In Vitro Release Medium

The stability of progesterone in NMP was continuously monitored until degradation was first detected, confirming that the drug remains stable in the 10% HP-β-CD-PBS release medium for at least 35 days. The stability was evaluated using the same sampling procedure and criterion as described in Section 4.2.4 (see Section 4.2.1 for detailed methodology).

### 2.3. Injectability Screening

Injectability represents a critical translational barrier for polymer-based injectable depots, directly determining clinical operability and industrial filling compatibility [27]. The ability to pass easily through a hypodermic needle and the performance during injection were studied.

As presented in Table 1, the greater the force required during injection, the larger the difficulties encountered during the injection process. Specifically, a formulation is deemed injectable when the required injection force is less than 50 N; when the force increases to 51–100 N, the injection begins to encounter some difficulties. As the force rises further to 100–130 N, injection becomes notably difficult, and it is recommended to replace the original needle with a larger one. When the required injection force exceeds 130 N, the injection operation becomes extremely challenging, and changing to a larger needle becomes the only way to continue.

To evaluate the injectability of progesterone gel injection, a total of 9 formulations were designed, with each formulation containing varying dosages of progesterone, and the polymer matrix is shown in Table 2.

During the preparation process, Formulation 5 and 9 displayed paste-like ultrahigh viscosity and failed to inject through any standard syringes, so they were eliminated from subsequent tests.

#### 2.3.1. Force–Displacement Analysis for Injectability Evaluation

**Figure 1:**
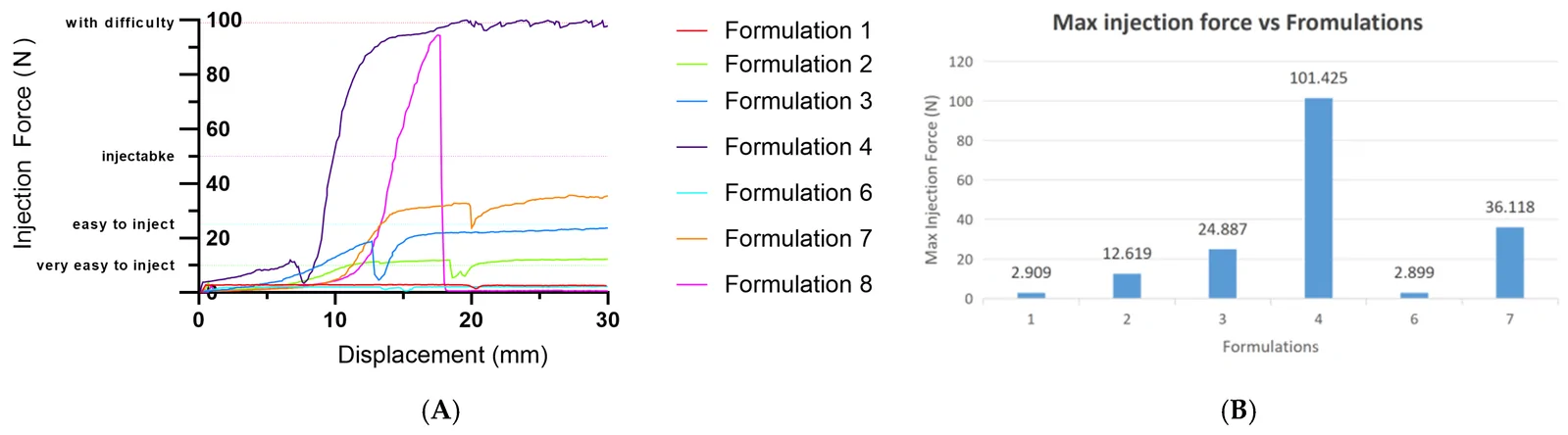
(**A**) Injection force–displacement curves of progesterone formulations during constant-speed injection (crosshead speed = 150 mm/min; injection volume = 2 mL). Since the plunger speed was held constant, time is linearly proportional to plunger displacement. The maximum force peaks indicate the highest resistance encountered during syringe extrusion. (**B**) Max injection force versus formulations.

The injectability of the seven injectable formulations was evaluated using an Auto Tensile Tester (PARAM MED-01) operating at a constant crosshead speed of 150 mm/min using a fixed injection volume of 2 mL in 23G needle. The force–displacement curves recorded during the injection process was illustrated in Figure 1A and the maximum injection force was shown in Figure 1B.

As shown in Figure 1A, injection force increased significantly with elevated progesterone loading or polymer concentration. To provide a comprehensive overview of the injectability across all formulations, the maximum injection force for each formulation was extracted and plotted in Figure 1B. The red curve (Formulation 1) and the light blue curve (Formulation 6) represent the groups with the lowest progesterone loading and the lowest polymer proportion, respectively. Both exhibit a maximum force less than 3 N, making the injection easy to perform for any operator.

As drug loading or polymer concentration increases, the injection force increases remarkably. When the progesterone loading reaches 40% (Formulation 4, purple curve), the maximum injection force approaches 100 N-a level that is practically unmanageable for medical staff. When the polymer proportion reaches 45% (Formulation 8, pink curve), the excessively high injection force causes the syringe needle to repeatedly detach from the syringe, injections were failed.

These results reveal critical formulation limits for clinical practicality: the polymer proportion should not exceed 35%, and progesterone loading should not exceed 30%, to ensure the injectability and practical usability of the products. Specifically, further increasing progesterone loading beyond 30% or polymer concentration beyond 35% led to a dramatic increase in injection force, eventually leading to failure in injection. In the meantime, the solubility limitation of progesterone in NMP is (187.91 ± 0.03 mg/mL), indicating that excessive drug loading results in a substantial undissolved solid phase that compromises injectability.

#### 2.3.2. Discharge Rate Analysis

The discharge rate is calculated by Equation (1), and the results are shown in Table 3.

As shown in Table 3, the discharge rate of each formulation exhibited notable variability that Formulations 5, 8, and 9 were not available to discharge. Formulation 1 achieved the highest discharge rate at 85.33%, there was a slight decreasing trend in discharge rate as the proportions of progesterone and polymer increased in Formulation 2, 4, 6, and 7. This observation aligns with the injection force data, suggesting that higher proportions of drug or polymer may compromise both injectability and discharge efficiency.

During the administration of the in situ forming depot formulation, the injected polymer–drug mixture tends to agglomerate and block the needle bore. This causes substantial drug retention within the syringe and needle, resulting in inaccurate dosing and compromised therapeutic efficacy. This not only causes inaccurate drug dosage administration; it also influences the therapeutic efficacy of the drug. Therefore, the drug discharge rate is a necessary factor that should be considered in the development of injectable formulations to ensure that the final pharmaceutical product can be injected into the body at a certain amount, thereby guaranteeing the final injected dose of the drug to achieve the desired pharmacological effect. It is evident that investigations into injectability are critical for both industrial manufacturing and clinical administration, and carry great forward-looking significance for the future commercial translation of lab-scale formulations.

The present data clearly demonstrated that both progesterone loading and PLGA polymer concentration were dominant viscosity-driving variables. The solubility of progesterone in NMP was determined to be 187.91 ± 0.03 mg/mL at 25 °C, confirming that in Formulation 5 (50% w/w P4), the drug loading far exceeded its solubility. During preparation, this formulation exhibited pronounced opalescence and visible particulate aggregation, indicating the presence of a substantial undissolved solid phase. Excess progesterone (50% w/w) generates dense drug aggregates that drastically elevate suspension viscosity, not only because the drug is undissolved, but also due to the excessively high solid fraction renders the suspension to become non-injectable. In highly concentrated suspensions, increasing solid loading dramatically elevates viscosity and yield stress—the minimum force required to initiate flow. When the particle concentration exceeds a critical threshold, strong inter-particle interactions lead to the formation of a particle network that exhibits paste-like rheology. Moreover, high-concentration suspensions are prone to needle occlusion caused by particle accumulation, bridging, and jamming at the needle orifice—a phenomenon where the solid phase locally accumulates to obstruct flow, leading to injection failure [28]. These effects are well-documented for concentrated suspension injections: increasing particle concentration and vehicle viscosity significantly increases the risk of clogging, with dense suspensions behaving like granular materials where density fluctuations create “arches” spanning the constriction that prevent further passage of particulate material [28]. While higher polymer or drug contents in PLGA/NMP in situ forming systems increase formulation viscosity and may compromise syringeability [29]. PLGA concentrations exceeding 35% w/w increase polymer chain entanglement within NMP solvent. For progesterone-specific PLGA delivery systems, lipophilic progesterone molecules tend to gather inside PLGA networks when excessively added, increasing inter-particle friction and extrusion resistance, which provides direct experimental support for the 30% maximum drug-loading threshold summarized in this study. This explained the failed administration performance of Formulation 5 and Formulation 9 in our preset matrix. Both scenarios produced unacceptably high injection force (>50 N) and low discharge efficiency, risking inaccurate clinical dosing and needle blockage during mass production.

The screened compositional window (progesterone ≤ 30% w/w; PLGA ≤ 35% w/w) balances high drug loading for therapeutic efficacy and low viscosity for smooth administration. The maximum discharge rate exceeding 82% within this range minimized residual drug waste in syringes, reducing production material loss and ensuring precise injected dose consistency—an essential requirement for pharmaceutical scale-up.

### 2.4. In Vitro Drug Release

On the basis of the preliminary formulation screening, the drug loading of progesterone in the formulation was set at 20%, and the proportion of the polymer was investigated of 15%, 20%, 30%, and 35% in NMP solution.

#### 2.4.1. Morphology of In Situ Gel Depot

**Figure 2:**
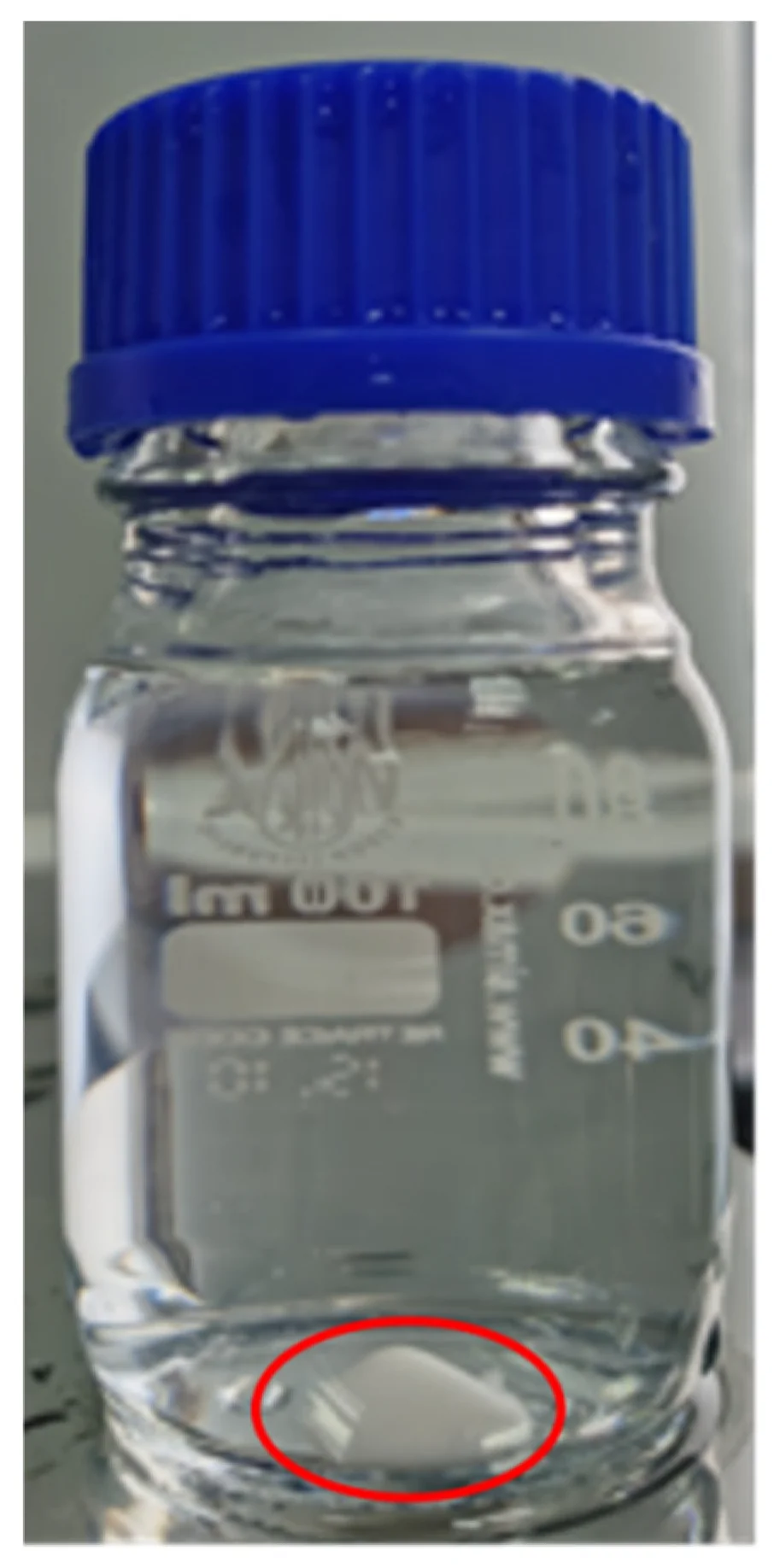
Morphology of the progesterone in situ gel after injection into release medium.

The morphology of the self-prepared progesterone gel formulation following syringe injection into the dissolution medium is presented in Figure 2 (the white object marked with a red circle). As observed, the formulation formed a stable, smooth ellipsoidal depot with a soft texture that remained sharp upon gentle pressure after immersion in the dissolution medium.

#### 2.4.2. In Vitro Release Profile

In vitro drug release experiments were performed using a water bath shaker at 37 °C, with a shaking speed of 60 rpm. Sampling was conducted at predetermined time points: 2, 4, 8, and 24 h, and 2, 3, 4, 5, 6, and 7 days post-injection. At each sampling time point, 50 μL of the release medium was withdrawn, followed by quantitative analysis via high-performance liquid chromatography (HPLC), according to the method described in Section 4.2.1. Additionally, the gel formulations in blue-cap vials were visually observed and photographed daily throughout the entire experimental period.

**Figure 3:**
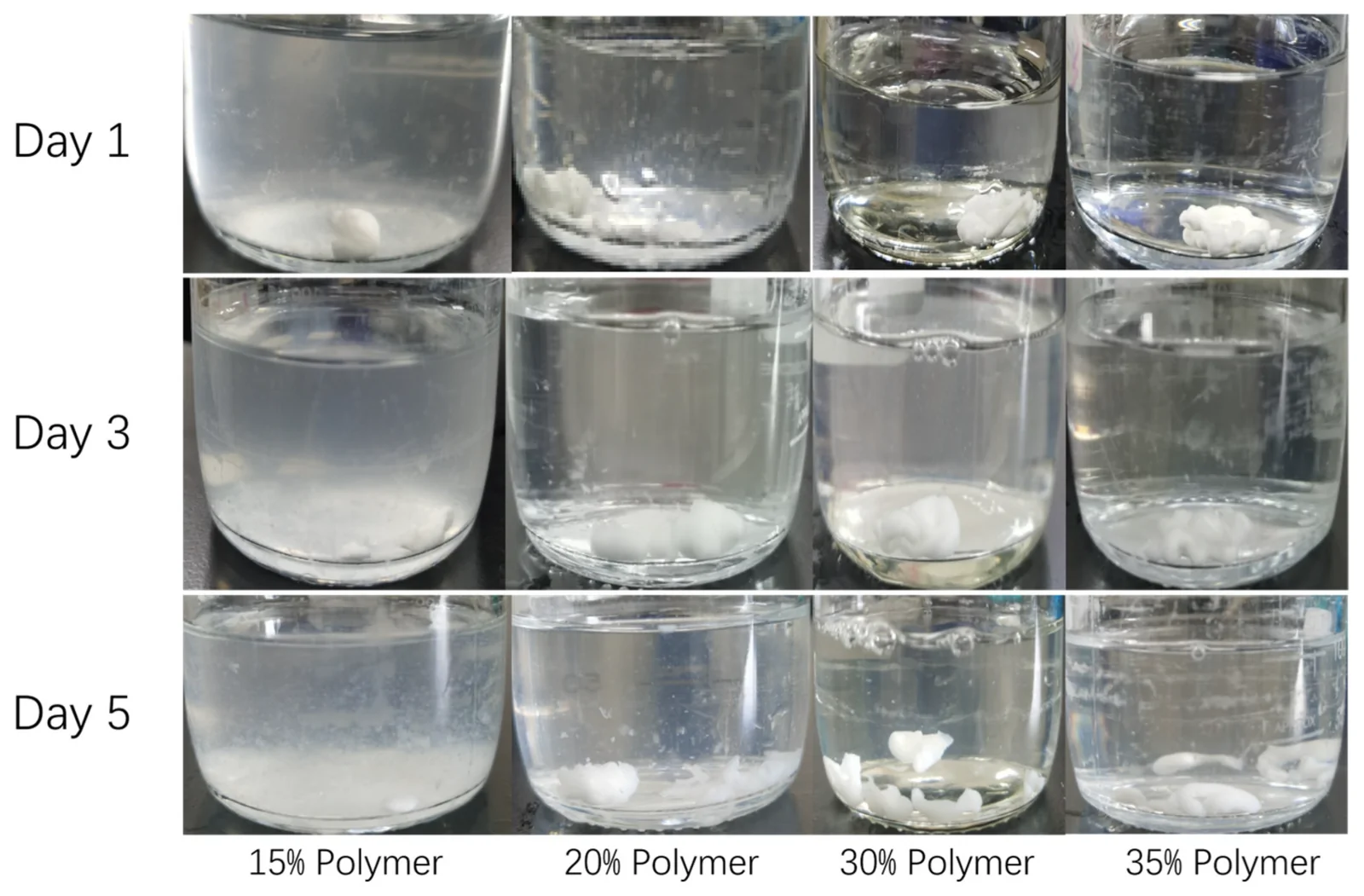
Appearance of progesterone gel depots with different PLGA concentrations in release medium on day 1, day 3, and day 5.

As shown in Figure 3, the stability of the gel injections in the in vitro release medium exhibited a strong dependence on polymer concentration.

For the formulation with 15% polymer, a stable drug reservoir failed to form following injection into the release medium. Turbidity was observed in the medium as early as 2 h post-injection. By day 3, the original depot had disintegrated into multiple small fragments, accompanied by a notable increase in medium turbidity. On day 5, only minute gel particles remained visible, and the entire release medium had become highly turbid.

**Figure 4:**
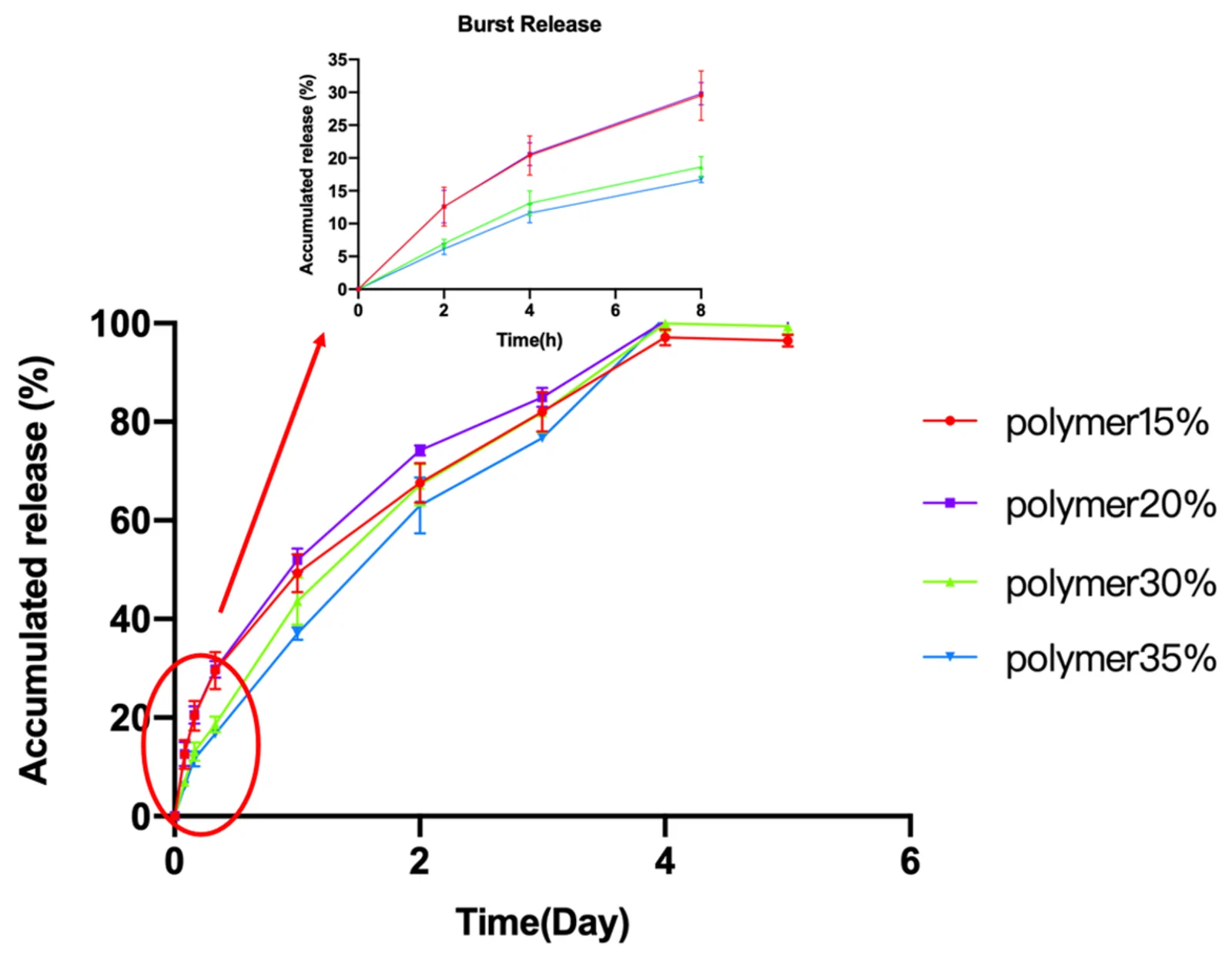
In vitro release and burst release curves of progesterone gel injection prepared with different PLGA concentrations. The smaller curves at the top of the graph represented the burst release profile within 8 h.

The in vitro release profiles of the four concentrations of polymer are shown in Figure 4 below:

As shown in the Table 4, formulations containing 15% and 20% polymer exhibited a distinct burst release phenomenon, with cumulative burst release rates reaching approximately 29.5% and 29.8%, respectively, at 8 h. In contrast, formulations with higher polymer contents (30% and 35%) demonstrated significantly reduced burst release, with cumulative values of only 18.61% and 16.74% at the same time point. Notably, despite these differences in initial burst release, all four formulations exhibited nearly identical overall release kinetics, achieving complete drug release by the 4th day. These findings clearly indicate that polymer concentration plays a critical role in modulating the initial burst release, while having negligible impact on the total duration of drug release.

Long-acting injections can release drugs in a stable and controllable manner, making in vitro release behavior a critical performance indicator. The drug release pattern of solvent-triggered PLGA in situ depots follows a triple-stage kinetic pathway: initial rapid solvent–water exchange triggers surface burst leakage, medium-term drug diffusion through matrix pore channels dominates release, and late-stage PLGA surface erosion continuously replenishes drug output [23,24]. Early excessive burst release is a core barrier to stable long-term hormone concentration maintenance, and our data verified PLGA concentration as an adjustable parameter to mitigate this defect without altering total release duration. Experimental in vitro release data showed that elevating PLGA proportion from 15% to 35% cut 8 h cumulative burst release from 29.51% to 16.74%; nevertheless, all tested groups achieved complete progesterone elution within four days regardless of polymer content difference. This two-way regulatory phenomenon can be explained by the disparity of solvent diffusion speed and post-injection three-dimensional depot microstructure. NMP possesses strong water miscibility; low-density PLGA networks cannot restrain solvent outward migration after subcutaneous injection, leading to fast matrix pore formation and quick escape of surface-adsorbed progesterone [21]. Higher PLGA fractions compact polymer chain arrangement, slow NMP efflux velocity, and form dense ellipsoidal depots with narrow, winding internal pores that extend water penetration paths and block rapid drug diffusion [25]. PLGA polymer matrix went through slow surface erosion rather than rapid degradation during the experiment period; thus, the polymer framework still maintained macroscopic structural integrity on day 5. The complete degradation of the PLGA matrix required a much longer period, far beyond the initial drug release time [30,31].

### 2.5. Histopathological Analysis of Subcutaneous Tissue

To assess the local tissue response to progesterone injectable gels with different polymer concentrations, histopathological examinations of four groups were performed on subcutaneous tissue samples collected from rats euthanized on day 7.

**Figure 5:**
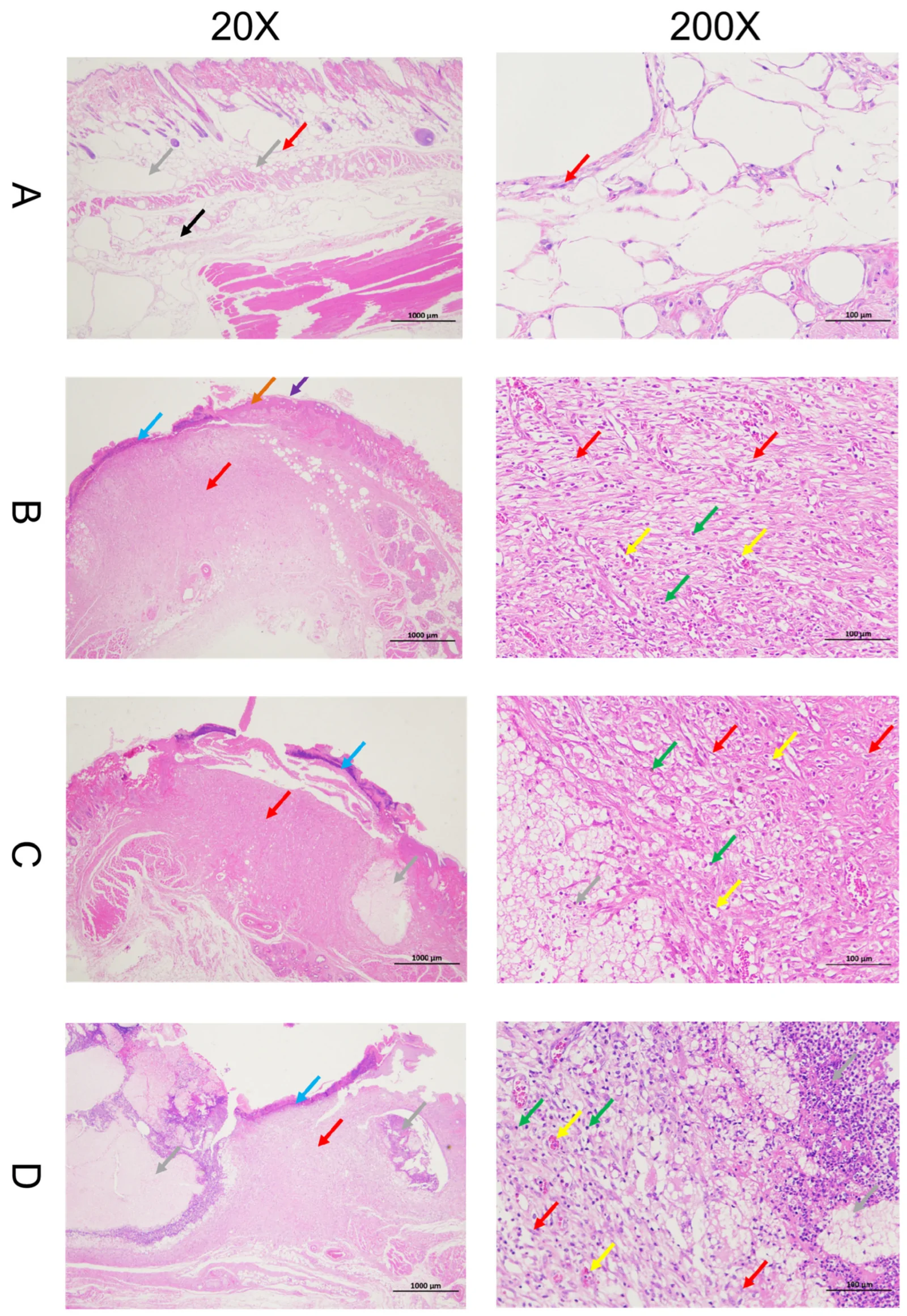
Histopathological images of subcutaneous injection sites (H&E staining, 20× and 200× magnification, and scale bar = 100 μm)). (**A**) Control group (commercially progesterone oil injection): Intact epidermis and dermis, mild connective tissue hyperplasia (red arrow), local edema (black arrow), varying sizes of fat cells (gray arrow), and small amount of inflammatory cell infiltration. (**B**) The 20% polymer group: Large area of eosinophilic material and necrotic cell debris (blue arrow), marked connective tissue hyperplasia (red arrow), abundant new blood vessels (yellow arrow), mild lymphocyte and granulocyte infiltration (green arrows); epidermal thickening around the lesion area (orange arrows); and hyperkeratosis and thickening of the stratum corneum (purple arrows). (**C**) The 30% polymer group: Epidermal loss, eosinophilic necrotic material (blue arrow), and local connective tissue hyperplasia (red arrow). focal eosinophilic material with scant necrotic debris and granulocyte infiltration (gray arrow), proliferation of fibroblasts and fibrocytes (red arrow), abundant new blood vessels (yellow arrow), and mild lymphocyte and granulocyte infiltration (green arrows). (**D**) The 35% polymer gel group: Local eosinophilic material with macrophage infiltration (gray arrow), mild connective tissue hyperplasia (red arrow), extensive eosinophilic necrotic material (blue arrow), large areas of eosinophilic material with abundant necrotic debris and granulocyte infiltration (gray arrow), marked connective tissue hyperplasia with fibroblast proliferation (red arrow), abundant new blood vessels (yellow arrow), and mild lymphocyte and granulocyte infiltration (green arrows).

Subcutaneous tissue samples were collected on day 7 after injection for hematoxylin and eosin (H&E) staining and pathological observation. The commercial progesterone oil injection was set as the control group. Pathological changes and semi-quantitative histological scores are shown in Figure 5 and Table 5.

#### 2.5.1. Control Group (Commercially Progesterone Oil Injection)

Histological analysis of control group skin revealed an intact epidermal architecture, including a well-defined stratum corneum (Figure 5A). The dermis exhibited a regular organization of collagen fibers and a uniform distribution of skin appendages, such as hair follicles and sebaceous glands. Within the subcutaneous tissue, adipocytes were arranged irregularly and displayed considerable size heterogeneity (gray arrows). Mild connective tissue hyperplasia was noted surrounding a minority of adipocytes (red arrows), concurrent with localized edema (black arrows) evidenced by loosely arranged connective tissue and vasodilation. Scattered infiltrates of lymphocytes and granulocytes were present. The absence of significant necrosis, marked inflammation, or tissue damage suggests a mild local tissue reaction to the injected commercial oil.

#### 2.5.2. The 20% Polymer Gel Group

In comparison to the control group, the 20% polymer gel group elicited more pronounced local tissue reactions (Figure 5B). The majority of specimens preserved an intact epidermis and dermis, characterized by well-organized collagen fibers and a typical distribution of skin appendages. Substantial regions of eosinophilic material were, however, evident within the subcutaneous tissue, associated with abundant necrotic cellular debris and granulocyte infiltration (blue arrows). Prominent connective tissue hyperplasia was observed in adjacent regions (red arrows), featuring proliferating fibroblasts and fibrocytes. Numerous newly formed blood vessels were also identified (yellow arrows), in addition to minor infiltrates of lymphocytes and granulocytes (green arrows). One sample, notably, displayed focal epidermal loss, extensive eosinophilic necrotic material (blue arrows), and effacement of the dermal and subcutaneous architecture by substantial connective tissue hyperplasia. Adjacent to the lesion, epidermal thickening, hyperkeratosis, and an increased stratum corneum were noted (orange and purple arrows). No significant edema was observed in any sample from this treatment group.

#### 2.5.3. The 30% Polymer Gel Group

Histological analysis of the 30% polymer gel group revealed moderate local tissue reactions (Figure 5C). The majority of specimens exhibited epidermal loss, with extensive regions of eosinophilic necrotic material evident in the subcutaneous tissue (blue arrows). This eosinophilic material was associated with scant necrotic cellular debris and granulocyte infiltration (gray arrows). Consistent with observations in the 20% group, prominent connective tissue hyperplasia (red arrows), proliferation of fibroblasts and fibrocytes, and abundant neovascularization (yellow arrows) were noted, accompanied by mild lymphocytic and granulocytic infiltration (green arrows). In one specimen, partial loss of the epidermis and dermis was observed, with the dermal layer replaced by substantial connective tissue hyperplasia. No significant edema was detected in this group.

#### 2.5.4. The 35% Polymer Gel Group

In the 35% polymer gel group, heterogeneous local tissue responses were evident (Figure 5D). Two specimens exhibited epidermal loss, extensive regions of eosinophilic necrotic debris (blue arrows), and substantial deposition of eosinophilic material within the subcutaneous tissue, accompanied by abundant necrotic cellular debris and granulocyte infiltration (gray arrows). Prominent connective tissue hyperplasia (red arrows), proliferation of fibroblasts/fibrocytes, and numerous newly formed blood vessels (yellow arrows) were also noted, in addition to mild infiltration of lymphocytes and granulocytes (green arrows).

#### 2.5.5. Histological Score

Histological scoring is a standard semi-quantitative method to evaluate in vivo biocompatibility and tissue response to implanted materials. In this study, as shown in Table 5, the control group showed a score of 0, indicating normal tissue architecture without any pathological changes. All polymer gel groups (20%, 30%, and 35%) exhibited a minimal histological score of 1, which corresponds to only mild, localized tissue reactions such as slight edema or sparse inflammatory cell infiltration, without evidence of severe inflammation, necrosis, or structural tissue damage. Notably, no dose-dependent increase in histological scores was observed across the tested concentrations. These findings collectively suggest that the polymer gels possess good biocompatibility in the local tissue microenvironment, as they did not elicit significant adverse pathological responses under the current experimental conditions. Although NMP is a biocompatible pharmacopeial excipient, the results indicated that NMP injections had potential mild local irritation.

We set commercially available progesterone oil injection as the control group and carried out 7-day subcutaneous implantation experiments on SD rats to conduct semi-quantitative histopathological scoring. The oil control group gained a score of 0, with nearly no pathological lesions, while all PLGA gel groups scored 1, accompanied by mild macrophage infiltration and slight connective tissue hyperplasia; no large-area tissue necrosis, abscess, or severe inflammatory cell aggregation was observed in all gel groups. The mild transient inflammation triggered by gel formulations mainly stems from fast outward diffusion of NMP after injection, which temporarily changes interstitial fluid osmotic pressure and recruits inflammatory cells [32]. Although oil injection brings mild short-term tissue reaction, abundant clinical and preclinical literature has recorded clinical drawbacks: persistent muscle soreness, sterile abscess risk, and two-week oil vehicle clearance time [4,33]. On the contrary, PLGA polyester material can be completely hydrolyzed into lactic acid and glycolic acid, which enter human metabolic circulation without permanent tissue residue, proving possible parenteral safety of this delivery platform [24,25]. No severe tissue damage was detected in all experimental groups, proving the basic parenteral safety of this delivery system.

It is worth noting that compared with the commercially available progesterone oil injection, NMP solvent poses greater potential local irritation risks, which should be fully considered in the early formulation design of similar in situ gel preparations. Subsequent screening of mixed solvent systems should be adopted to further reduce NMP-induced stimulation.

## 3. Discussion

### 3.1. Formulation Rationale and Technology Selection

Progesterone is characterized by extremely low aqueous solubility (0.007 mg/mL at 25 °C) and high lipophilicity (logP = 3.87), which impose specific constraints on formulation design. The clinical requirement for sustained exposure of progesterone determines a delivery system capable of maintaining therapeutic levels over multiple days. The selection of PLGA/NMP in situ forming gel was therefore based on a comparative evaluation of available technologies against these specific requirements.

Comparison with oil-based intramuscular injections: Progesterone-in-oil injections remain the most widely used parenteral formulation but require daily or every-other-day administration and are associated with cumulative local tissue irritation, including soreness, sterile abscess formation, and allergic reactions [11,13]. In contrast, PLGA-based in situ forming systems are administered as a single injection and release the drug over an extended period, substantially reducing the frequency of administration and eliminating the cumulative oil burden associated with repeated dosing [22]. Upon injection, the organic solvent (NMP) diffuses into the surrounding tissue fluid, triggering precipitation of the polymer into a solid depot. The PLGA matrix then undergoes hydrolytic degradation via ester bond cleavage, yielding lactic acid and glycolic acid as primary degradation products, which are ultimately metabolized to carbon dioxide and water and eliminated from the body [25,31].

Comparison with PLGA microspheres: PLGA microspheres are a well-established biodegradable platform for sustained delivery, with multiple FDA-approved products on the market, including Lupron Depot (leuprolide), Sandostatin LAR (octreotide acetate), Risperdal Consta (risperidone), and Vivitrol (naltrexone) [34]. However, the manufacturing process for PLGA microspheres is inherently complex. The traditional emulsion-solvent evaporation method involves multiple steps—including emulsification, solvent extraction, hardening, washing, and lyophilization—each requiring precise control [34]. In contrast, PLGA/NMP in situ gels require only dissolution of the polymer in the organic solvent followed by drug dispersion—a substantially simpler process [16]. This processing advantage, however, does not necessarily translate to superior product performance. PLGA microspheres, despite their complex manufacturing requirements, are pre-formed particles that can be thoroughly characterized—including particle size, drug loading, morphology, and porosity—before being filled into final containers, enabling well-established quality control frameworks [34]. In situ gels, by contrast, are formulated as low-viscosity solutions and only form the final solid depot upon injection, which limits the extent of pre-administration characterization of the depot structure [16]. Moreover, PLGA-based in situ gels face the well-recognized challenge of high initial burst release, typically occurring within 24 h post-administration, which can lead to drug toxicity and local tissue irritation [35]. For progesterone formulations, cost is a particularly relevant factor, as conventional progesterone-in-oil injections are cheap hormonal preparations in market. The significantly higher manufacturing costs of PLGA microspheres may limit their clinical and commercial viability for progesterone therapy; meanwhile, in situ gels offer a more economically feasible approach to developing a long-acting progesterone product based on their much simpler manufacturing process.

Comparison with thermosensitive hydrogels: Thermosensitive polymers such as Poloxamer have been investigated as drug delivery carriers, offering the advantages of an aqueous vehicle and sol–gel transition at body temperature. However, studies have shown that in poloxamer-based progesterone thermosensitive in situ gels, the optimal formulation contains only 4.0% (w/w) progesterone [36]. Even with nanosuspension strategies to improve drug dispersion, the progesterone concentration in thermosensitive gel matrices remains limited to approximately 6 mg/mL [37]. PLGA/NMP formulations, by contrast, are proved to accommodate substantially higher progesterone loadings (up to 35% (w/w)) in our study. Given the high drug-loading requirement for long-acting progesterone preparations, such low loading capacity of thermosensitive hydrogels cannot meet this demand, rendering them unsuitable for this type of long-term delivery system.

Comparison with SAIB-based systems: Sucrose acetate isobutyrate (SAIB)-based in situ forming implants represent a viable alternative to PLGA-based depot systems. SAIB is a highly viscous hydrophobic liquid that forms a solid-like depot upon injection, with drug release governed primarily by diffusion rather than polymer degradation, and can achieve release durations ranging from several days to weeks via formulation optimization [38]. However, SAIB release modulation relies primarily on formulation variables such as solvent type, additive composition, and drug loading, and the system is known to exhibit undesirable burst release. In contrast, PLGA enables adjustment of release kinetics through multiple parameters, including polymer molecular weight, lactide-to-glycolide ratio, and polymer concentration [25,31]. The current study developed a progesterone depot formulation with a release duration of several days; however, clinical requirements may vary across different treatment time frames. Compared with SAIB, PLGA allows straightforward modification of its inherent material properties to systematically modulate the release period. This superior intrinsic tunability renders PLGA a more flexible matrix for the iterative development of progesterone long-acting formulations, which constitutes the primary rationale for selecting PLGA in this study.

Consideration of biodegradable versus non-biodegradable polymers: Beyond the specific comparisons above, the choice of PLGA also reflects a fundamental preference for biodegradable materials over non-degradable alternatives (such as silicone elastomers, polyethylene, or polyurethane). Although non-degradable polymeric implants are capable of achieving long-term controlled release of drugs, they remain intact in vivo after drug exhaustion and require surgical removal, which inevitably increases patient physical burden and raises the risk of local inflammation and infection. In the event of complications or severe adverse effects, non-degradable implants could be removed through surgical intervention which could terminate future drug exposure [39]. In contrast, PLGA undergoes gradual and sustained hydrolytic degradation to produce lactic acid and glycolic acid monomers. These endogenous metabolites are further eliminated from the body via the tricarboxylic acid cycle in the form of carbon dioxide and water, ensuring excellent biosafety and in vivo clearance [25]. However, if in an emergency, the depot cannot be immediately removed and must be left to degrade naturally [39]. In order to minimize the impact of this risk as much as possible, RESOMER^®^ RG 501 H was selected among the commercially available PLGA products because it was the fastest degrading PLGA readily obtainable from Evonik at the time of procurement, with an official announced maximum degradation time of 3 months. Patients receiving progesterone supplementation do not require an additional procedure to remove the delivery system after the treatment is completed.

### 3.2. Key Formulation Parameters and Mechanistic Interpretation

The systematic screening of progesterone formulations in this study revealed two critical composition thresholds: progesterone loading ≤30% w/w and PLGA concentration ≤35% w/w. These thresholds governed injectability, burst release, and overall formulation feasibility through distinct but interrelated mechanisms.

Injectability and the 50 N threshold. The injectability of the formulations, evaluated by injection force and discharge rate, showed a clear composition-dependent pattern. Formulations with progesterone loading of 30% or less and PLGA concentration of 35% or less exhibited injection forces below 50 N, with discharge rates exceeding 79%. When progesterone loading reached 40% (Formulation 4), the maximum injection force approached 100 N-a level practically unmanageable for clinical administration. At PLGA concentrations of 45% and above (Formulations 8 and 9), injection failed due to excessive resistance that repeatedly detached the needle from the syringe. The 50 N threshold used in this study follows the injectability classification reported by Rungseevijitprapa and Bodmeier, who defined injection forces of 26–50 N as “injectable” and forces exceeding 50 N as progressively difficult for manual administration. This classification has been adopted in the evaluation of injectable polymer-based depot systems and reflects the practical limits of manual syringe operation by healthcare providers [27,40]. This classification reflects the practical limits of manual injection by healthcare providers and serves as a clinically relevant benchmark for formulation feasibility.

PLGA concentration and burst release: The in vitro release data demonstrated that elevating PLGA concentration from 15% to 35% reduced the 8 h cumulative burst release from approximately 29.8% to 16.74%. This observation is consistent with the established understanding of PLGA-based in situ forming systems: higher polymer concentration increases solution viscosity, which slows the outward diffusion of NMP into the surrounding aqueous medium. The resulting slower phase separation leads to the formation of a denser polymer network with smaller and more tortuous pores, thereby extending water penetration pathways and restricting rapid drug diffusion through the matrix. In the early stage of release, progesterone—being a small lipophilic molecule—is released primarily through diffusion via water-filled pores formed during the phase inversion process. The dense polymer matrix acts as a diffusion barrier that limits the initial escape of surface-associated drug. This interpretation is supported by the tri-phasic release mechanism described by Fredenberg et al., where the initial burst phase is governed by diffusion of surface-associated or easily accessible drug molecules, followed by diffusion through pore channels and later polymer erosion [31].

Release duration and clinical matching: All tested formulations completed progesterone release within 4 days under in vitro sink conditions. This relatively short release duration may appear inconsistent with the longer degradation time of PLGA, but the two phenomena are governed by different mechanisms. In PLGA-based systems, the initial drug release (days to weeks) is primarily diffusion-controlled through water-filled pores, whereas polymer erosion—which governs the later-stage release of encapsulated drug from the matrix—occurs over weeks to months [39]. The PLGA used in this study (RG of 501 H, Mw of ~7000 Da, and inherent viscosity of 0.12 dL/g) has a relatively low molecular weight, which accelerates hydration and pore formation but does not imply accelerated matrix erosion. Thus, the 4-day release reflects the diffusion of progesterone through the porous depot structure before extensive polymer degradation occurs.

This release duration falls within the intended design scope for this formulation. Given the high daily dose requirements of progesterone for luteal phase support, the initial objective was to develop a formulation capable of sustaining release over several days—rather than weeks or months—to reduce dosing frequency from daily injections to, ideally, a weekly administration schedule. The 4-day in vitro release profile is consistent with this objective, as it represents a substantial extension compared with conventional oil-based injections. However, the precise in vivo release duration cannot be directly inferred from the in vitro data, as the solubilizing effect of 10% HP-β-CD in the release medium may accelerate drug release relative to the physiological environment. The actual release kinetics in vivo—and the corresponding dosing interval—will require confirmation through pharmacokinetic studies in appropriate animal models.

### 3.3. Release-Medium Selection and In Vitro Release Considerations

Progesterone is a highly lipophilic drug with poor water solubility, requiring a solubilizing agent in the release medium to maintain sink conditions. Cyclodextrins was selected because it provided sufficient solubilizing capacity to maintain sink conditions throughout the in vitro release study, and it is necessary for obtaining reproducible release data for formulation comparison and quality control [41].

It is reported that higher cyclodextrin concentrations can reduce the free fraction of drug available for permeation or diffusion when used as solubilizing agents in in vitro media [41,42]. The cyclodextrin concentration should be kept at the minimum level that provides sufficient solubilization in order to avoid unnecessarily reducing the free drug concentration gradient that drives drug diffusion from the depot. Accordingly, 10% HP-β-CD was chosen as the minimum effective concentration: it provides sufficient solubilizing capacity (7.6 mg/mL) to maintain sink conditions without using excess cyclodextrin.

Beyond its solubilizing function, HP-β-CD has been increasingly recognized as a useful component in in vitro release media for establishing IVIVC. Aihara et al. [43]. investigated the concentration-dependent effects of HP-β-CD on BCS Class II drugs (danazol and albendazole) and demonstrated that HP-β-CD significantly increases the intrinsic dissolution rate under dissolution-rate-limited conditions, and the resulting in vitro permeation profiles can correlate with in vivo absorption outcomes. These findings indicate that HP-β-CD-containing media, while non-physiological, can serve as a predictive tool when the dissolution step is the rate-limiting factor governing drug absorption.

This approach has limitations. First, HP-β-CD binds to the drug and reduces the free fraction, creating a tradeoff between solubility and permeability [42]. Second, the rate-limiting step may differ between in vitro and in vivo systems [43]. Third, the static in vitro setup cannot mimic the dynamic in vivo environment, such as fluid turnover, enzymes, and tissue responses. Therefore, while the HP-β-CD medium is practical for formulation screening and quality assurance, its predictive ability should be confirmed by pharmacokinetic studies.

### 3.4. In Vivo Consideration, Comparative Evaluation, and Study Limitations

The present study is a formulation screening investigation focused on establishing the composition-performance relationship of PLGA/NMP-based progesterone depots in a controlled in vitro setting. Several factors should be considered when extrapolating these findings to the in vivo context. The injection procedure—including injection speed, needle gauge, and formulation volume—can influence depot morphology and solvent exchange kinetics, which in turn affect burst release and subsequent drug release [39]. Additionally, the local tissue environment at the injection site—such as vascular perfusion, tissue water content, and lipid composition—may influence NMP diffusion and PLGA precipitation. It has been reported that the syringe-needle assembly and injection rate can influence the shape of the in situ forming implant, and variations in implant geometry significantly affect drug release kinetics by altering the surface-to-volume ratio [44]. Accordingly, in both in vivo and in vitro experiments, the implant should carefully be formed as an intact and cohesive depot. Especially in the animal study, the injection was gently shaped immediately after administration to form a complete shape, thereby minimizing shape-related variability in drug release.

Progesterone’s high lipophilicity (logP 3.87) also suggests possible partitioning into adipose tissue, which could create a secondary drug reservoir and alter release kinetics compared with the aqueous in vitro medium. Furthermore, PLGA degradation and drug release in vivo may differ from in vitro observations due to enzymatic activity, inflammatory responses, tissue ingrowth, and non-sink conditions. This would be used in the design of subsequent pharmacokinetic investigations, which will be conducted with lead formulations identified from this screening. These considerations do not diminish the value of the present in vitro screening; rather, they define the scope of this study and inform the design of subsequent pharmacokinetic investigations.

NMP-induced local tissue response: Histopathological evaluation on day 7 following subcutaneous injection revealed that all PLGA gel groups (20%, 30%, and 35% polymer) elicited mild local tissue reactions with a histological score of 1, whereas the commercial oil injection control group scored 0, indicating nearly normal tissue architecture. The inflammatory response itself is attributed to the rapid diffusion of NMP into surrounding tissue fluid upon injection, which may cause transient changes in local osmolality and recruit inflammatory cells [39]. PLGA degradation products—lactic and glycolic acids—may also lower local pH, potentially contributing to a mild foreign-body reaction [31]. This observation confirms that the NMP-containing PLGA gels induce a mild but detectable inflammatory response. This study was designed as a proof-of-concept investigation, and the histopathological assessment was intentionally incorporated at this early stage (before extensive formulation optimization), which is meaningful to prospectively identify potential safety concerns that might arise in later clinical translation. Identifying this issue at the screening stage is an advantage: strategies can be incorporated into subsequent formulation development to avoid the more costly and time-consuming changes in advance. Strategies being considered to reduce NMP-related irritation include (i) exploring mixed solvent systems that combine NMP with less irritating co-solvents (e.g., ethanol, dimethyl sulfoxide, or PEG-400) to reduce the NMP content while maintaining polymer solubility and injectability; (ii) screening drug loading to minimize injection volume; and (iii) evaluating alternative, lower-irritation solvents for PLGA dissolution.

## 4. Materials and Methods

### 4.1. Materials

Micronized progesterone (USP-grade, batch No. HTT190903) was manufactured by Humanwell Healthcare (Group) Co., Ltd. (Wuhan, Hubei, China). Poly(D,L-lactide-co-glycolide) (RESOMER^®^ RG 501 H, lactide:glycolide = 53:47 mol%, acid-terminated, Mw ≈ 7000 Da by GPC, Mn ≈ 2300 Da, PDI ≈ 2.5, inherent viscosity of 0.12 dL/g (0.1% in chloroform at 25 °C), acid number 20 mg KOH/g, Tg 36 °C) was purchased from Evonik Specialty Chemicals (Shanghai) Co., Ltd. (Shanghai, China). N-Methyl-2-pyrrolidone (NMP, 99.5%, HPLC-grade) and other organic solvents (analytical-grade) were supplied by Sigma-Aldrich (St. Louis, MO, USA). Hydroxypropyl-β-cyclodextrin was acquired from Xi’an Deli Biochemical Co., Ltd. (Xi’an, China). Phosphate-buffered saline (PBS, pH 7.4, 0.01 M) was prepared in-house according to the Chinese Pharmacopoeia formulation: 4.303 g of anhydrous disodium hydrogen phosphate (Na_2_HPO_4_) and 1.179 g of potassium dihydrogen phosphate (KH_2_PO_4_) were dissolved in distilled water to a final volume of 1000 mL, and the pH was adjusted to 7.4 at 25 °C if necessary (all analytical-grade, Sinopharm Chemical Reagent Co., Ltd., Shanghai, China). Syringes and needles were purchased from Shanghai Kindly Medical Instruments Co., Ltd. (Shanghai, China). Commercial progesterone oil injection (1 mL:20 mg) was manufactured by Tianjin Pharmaceuticals hanGroup Co., Ltd. (Tianjin, China). GraphPad Prism (version 10.1.2 for Windows, GraphPad Software, Boston, MA, USA) and WPS Office (version 12.1.0.28043, Kingsoft Corporation, Zhuhai, China) were used for data processing, statistical analysis, and figure preparation.

### 4.2. Methods

#### 4.2.1. HPLC Method Validation for Progesterone Determination

Progesterone concentrations were quantified using a Shimadzu LC-20A HPLC system with LabSolutions software (Shimadzu Corporation, Kyoto, Japan), equipped with a SIL-20A autosampler, a CBM-20A system controller, and an SPD-20A UV–Vis detector, controlled by LabSolutions software (version 5.92, Shimadzu Corporation, Kyoto, Japan). Chromatographic conditions: Inertsil ODS-3 column (5 μm, 4.6 × 250 mm, GL Sciences, Tokyo, Japan); column temperature, 35 °C; mobile phase, methanol–acetonitrile–water (25:35:40, v/v/v); flow rate, 1.0 mL/min; detection wavelength, 241 nm; and injection volume, 10 μL. The calibration curve for progesterone was linear over the concentration range of 2–600 μg/mL, with a regression equation of y = 33620x − 5275.6 and a correlation coefficient of R^2^ = 0.999 (n = 6). The LOD and LOQ were 0.98 μg/mL and 2.97 μg/mL, respectively. Recovery rates were 98–102%, with RSD < 2.0%, and resolution between progesterone and degradation product peaks was >4.0, confirming accuracy, reproducibility, and system suitability for progesterone quantification.

#### 4.2.2. Preparation of Injectable Progesterone Solution

The preparation of an injectable gel delivery system for progesterone involved several key steps, including steps to screen polymer swelling; micronized progesterone dispersion; the formation of a stable, homogeneous solution; and, at last, inject into injection site. The initial step in developing such a system was the precise weighing and complete dissolution of the polymer. The polymer, serving as the matrix for the injectable gel, was accurately weighed and then incrementally introduced into a suitable solvent system, typically NMP, under continuous magnetic stirring. NMP was chosen because (1) NMP has excellent solvency for both PLGA and progesterone; (2) it has a favorable water-miscibility that allows rapid solvent exchange and in situ gel formation; (3) it has been widely used in FDA-approved in situ forming depot products (e.g., Eligard^®^); and (4) it exhibits acceptable local tolerability compared to alternative solvents, such as DMSO or 2-pyrrolidone. The stirring process was maintained at a controlled speed for a minimum of 10 min, which was crucial for the dissolution of the polymer. Following this, the mixture was allowed to equilibrate at 4 °C overnight, which allowed the complete polymer swelling and hydration, yielding a homogeneous and transparent polymer solution. The resulting polymer solution should be optically clear and free from undissolved particles, indicating successful and uniform polymer solution preparation. The micronized progesterone was carefully added to the polymer solution, and the mixture was subjected to high-speed homogenization using an advanced electric homogenizer (at approximately 8000–10,000 rpm) for 5 min, aiming to break down any aggregates of progesterone and distribute more uniformly throughout the polymer solution. Following the homogenization, continuous stirring was maintained for a minimum of 30 min under controlled conditions, and the progesterone injectable gel was obtained. A total of nine formulations (as shown in Table 2) with varying progesterone (10–50%, w/w) and polymer (15–55%, w/w) concentrations were prepared for screening.

#### 4.2.3. Determination of Equilibrium Solubility

Based on the literature review and the previous work of our laboratory on the key common technology platform of “biodegradable long-acting drug delivery system”, HP-β-CD PBS solution was selected as the in vitro release medium, and the ratio of HP-β-CD was investigated at 6%, 10%, and 15%. Excess progesterone was added to the glass bottles containing 6%, 10%, and 15% HP-β-CD PBS solution. The solution bottles were placed in the incubators at a temperature of 37 °C, 100 rpm. Then, 1.5 mL of supernatant from each bottle was collected at regular time intervals of 8 h, 24 h, 48 h, 96 h, and 168 h, and was immediately filtered through a 0.45 µm hydrophilic PTFE syringe filter (Anpel Laboratory Technologies (Shanghai) Inc., Shanghai, China); in the meantime, syringes, filters, and any containers should be preheated to 37 °C. Subsequent filtrate was diluted 4 times with acetonitrile and analyzed by HPLC, and the concentration of progesterone in each solution was calculated. When the relative percentage difference (|C_1_ − C_2_|/C_1_ × 100%) between two consecutive sampling time points was less than 2%, the concentration (Av, SD) was assumed as the equilibrium solubility of progesterone.

#### 4.2.4. Evaluation of Progesterone Stability in In Vitro Release Medium

The stability of progesterone in the selected 10% HP-β-CD/PBS release medium was evaluated under the in vitro experimental condition (37 °C, consistent with the temperature used in in vitro release studies). Supernatants were carefully collected from each equilibrated solubility sample and immediately subjected to micro-filtration through a 0.45 µm microporous membrane to remove any undissolved drug particles or excipient aggregates. The clarified filtrate was then quantitatively transferred into pre-cleaned 25 mL stoppered glass bottles. These vessels were subsequently positioned within a temperature-controlled incubator, maintained at 37 ± 0.5 °C, to faithfully mimic human physiological temperature. A 1 mL sample was collected at predefined temporal intervals until degradation was observed, at which point the experiment was terminated. Immediately upon collection, each sample underwent centrifugation at 8000 rpm for 10 min. The supernatant was analyzed by HPLC, all operations were performed in triplicate (n = 3), and results are expressed as mean ± SD.

#### 4.2.5. Evaluation of Injectability

Injectability was evaluated by two parameters: injection force and discharge rate. The ability to pass through a hypodermic needle and the performance during injection were studied.

##### Injection Force Measurement

**Figure 6:**
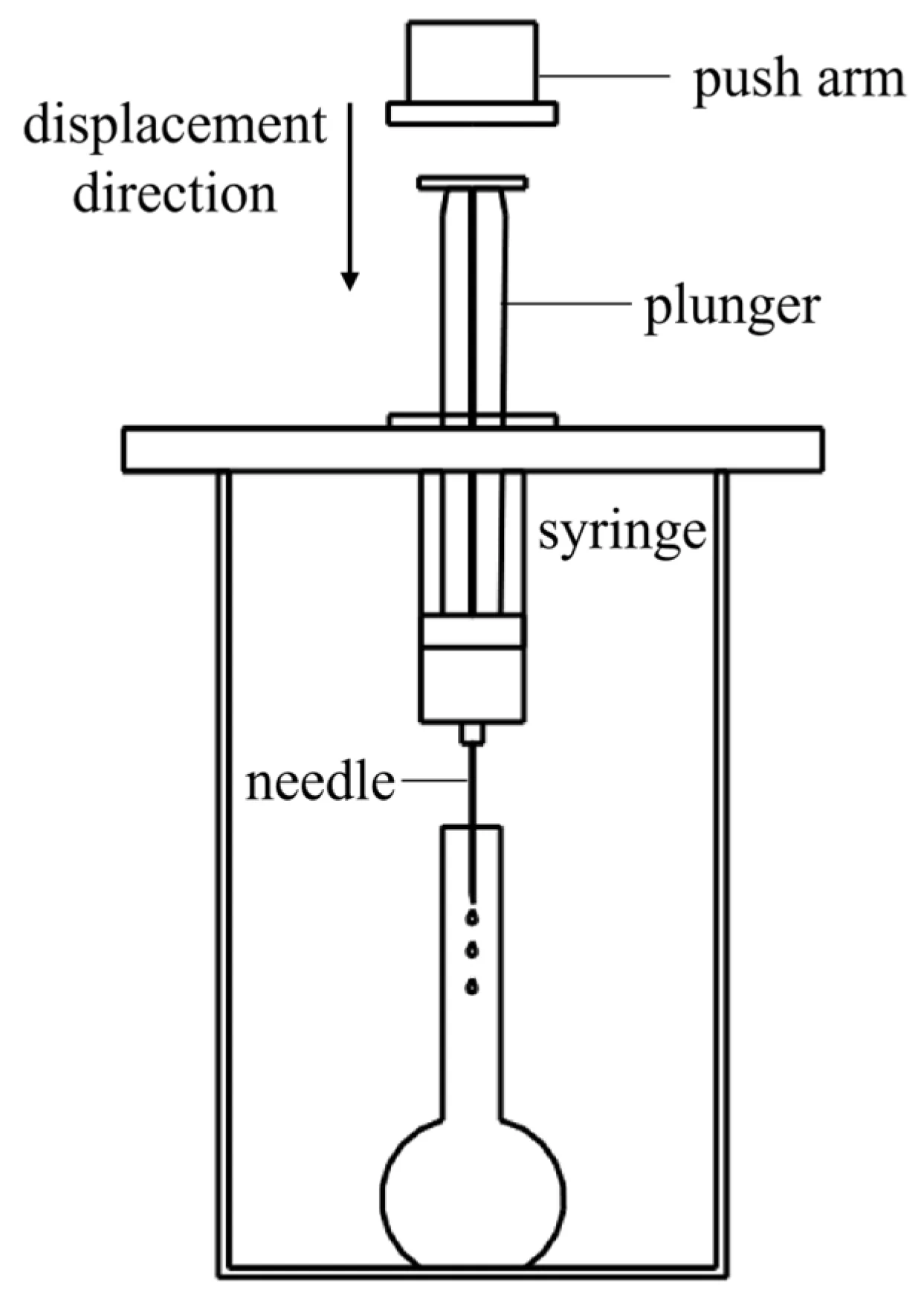
Instrumental setup for injection force measurements. The system consisted of a push arm, plunger, fixed syringe, and needle used to simulate manual injection and record force–time profiles.

The force tests were conducted using the Tensile Tester (PARAM MED-01, Labthink Instruments Co., Ltd., Jinan, Shandong, China). The instrument operated by pushing the plunger to move downward, the relative movement allowed the force sensor to record force and displacement changes throughout the test, thereby calculating various mechanical performance parameters. The fixed syringe was filled with 2 mL of formulation and fitted with a 23G needle, and then it was pushed by flat crosshead plunger at a constant speed (150 mm/min) to simulate the manual injection process at 25 °C (controlled room temperature). The time-vs.-force curve during this process was recorded, while the frictional force inherent to the syringe was negligible (about 0.6 N with 23G needle). The instrumental setup for the injection force measurements is shown in Figure 6.

##### Discharge Rate

Drug residue in syringes and needles after injection was measured via the weight loss method. The syringe containing suspension was weighted (W_0_), and the weight of syringe was recorded after injection (W_1_). The discharge rate was calculated based on the residual weight to the original loaded weight in the syringe. The formula is as follows (Equation (1)):

WD (%) = (1 − W_1_/W_0_) × 100% (1)

WD: The discharge rate of the formulation, %;

W_1_: The weight of the residual syringe, g;

W_0_: The weight of the original syringe loaded suspension, g.

#### 4.2.6. In Vitro Drug Release Study

A volume of 1 mL of the prepared progesterone injectable gel was precisely injected, via a 1 mL disposable syringe fitted with a 23-gauge × 32 mm needle, into a glass vial (inner diameter = 12 mm) containing 100 mL of 10% hydroxypropyl-β-cyclodextrin (HP-β-CD) phosphate-buffered saline (PBS). In order to avoid separation of the suspension, the progesterone long-acting gel was used immediately after prepared in the in vitro release. The gel was slowly injected into the in vitro release medium of 10% HP-β-CD PBS. During the injection, the needle of the syringe should be kept close to the bottom of the blue-cap bottle, and shaking of the arm should be avoided as much as possible during injection to ensure that the injection is performed at the same position which could simulate the situation during real injection in body. The vial was then transferred quickly to a laboratory incubator maintained at 37 °C and shaken at a constant speed of 60 rpm for the predetermined experimental duration. Aliquots of the release medium (50 μL) were withdrawn at preset time intervals: 2, 4, 8, and 24 h, followed by 2, 3, 4, 5, 6, and 7 days. Each collected sample was immediately transferred to a centrifuge tube for subsequent processing. The concentration of progesterone in the PBS samples was quantified using a Shimadzu high-performance liquid chromatography (HPLC) system at a wavelength of 241 nm. All experiments were conducted in triplicate, with results expressed as mean values ± standard deviation (SD), n = 3.

#### 4.2.7. Histopathological Analysis

Female SPF-grade Sprague–Dawley (SD) rats (200 ± 10 g, 6–8 weeks old) were supplied by Shanghai Cipro Bikai Laboratory Animal Co., Ltd. (Shanghai, China). All animal procedures were approved by the Ethics Committee of Shanghai Institute for Planned Parenthood Research (approval No. 2019-33).

To assess the local tissue response to progesterone injectable gels with different polymer concentrations, histopathological examinations of four groups (n = 2) were carried out by Servicebio Technology Co., Ltd. (Wuhan, China), and tissue samples were sourced from rats euthanized on day 7. All tissue samples were fixed in 4% paraformaldehyde and processed following standard histological protocols (dehydration, embedding, sectioning, and staining). Histopathological observations were performed using a Nikon Eclipse Ci-L upright light microscope (Nikon Corporation, Tokyo, Japan). Microscopic examination of tissue sections or digital slides at varying magnifications enabled a detailed assessment of pathological alterations, such as congestion, hemorrhage, edema, degeneration, necrosis, hyperplasia, fibrosis, organization, granulation tissue formation, and inflammatory changes. A descriptive account should accompany the observations to emphasize distinctions among samples. Representative pathological regions should be captured as images and annotated with arrows.

## 5. Conclusions

In this study, we developed and screened a series of PLGA/NMP-based injectable in situ forming gels for sustained progesterone delivery. Through a nine-formulation matrix with varying progesterone loadings (10–50% w/w) and PLGA concentrations (15–55% w/w), we established two critical composition thresholds that govern formulation feasibility: progesterone loading should not exceed 30% w/w, and PLGA concentration should remain at or below 35% w/w. Formulations within this optimal composition range exhibited acceptable injection forces (<50 N) and discharge rates exceeding 79%, while increasing PLGA concentration from 15% to 35% progressively reduced the 8 h burst release from 29.8% to 16.74%, indicating the formation of a denser polymer network that slows solvent exchange and drug diffusion. All formulations released progesterone within 4 days under in vitro condition, a prolonged administration interval when compared to conventional oil-based injections. Histopathological evaluation on day 7 showed mild local tissue reactions (score 1) for all PLGA gel groups, indicating acceptable biocompatibility irritation attributable to NMP diffusion.

These findings identified a potential formulation window that balances injectability, release kinetics, and local tissue tolerance for a progesterone sustained-release injectable. The mild NMP-related irritation observed at this screening stage provides valuable early insight for subsequent formulation development. Future study will focus on exploring mixed solvent systems to reduce the irritation, and efforts will also focus on establishing in vivo pharmacokinetics and release behavior.

## Figures and Tables

**Table 1:** Classification of injectability based on injection force.

Injection Force (N)	Injectability
0–10	Very easy to inject; the needle size can be reduced if needed
11–25	Easy to inject
26–50	Injectable
51–100	There are some difficulties with the injection
100–130	Injection difficult; should change the lager needle head
>130	It is very difficult to inject; you have to change to a bigger needle head

**Table 2:** Composition of nine progesterone in situ gel formulations (%, w/w).

	Injectability Test								
	F1	F2	F3	F4	F5	F6	F7	F8	F9
wt.% in N-methyl-2-pyrrolidone									
Progesterone	10	20	30	40	50	20	20	20	20
PLGA 501H (lactide:glycolide = 53:47)	20	20	20	20	20	15	35	45	55
Solids	30	40	50	60	70	35	55	60	75
wt.% based on solids									
Progesterone	33.3	50.0	60.0	66.7	71.4	57.1	36.4	30.8	26.7
PLGA 501H (lactide:glycolide = 53:47)	66.7	50.0	40.0	33.3	28.6	42.9	63.6	69.2	73.3
ratio based on solids									
Progesterone	1	1	1	1	1	1	1	1	1
PLGA 501H (lactide:glycolide = 53:47)	2.00	1.00	0.67	0.50	0.40	0.75	1.75	2.25	2.75

**Table 3:** Discharge rate of progesterone gel formulations.

Formulation	Discharge Rate (100%)
1	85.33
2	82.25
3	82.79
4	78.75
5	Not available
6	82.96
7	79.91
8	Not available
9	Not available

Note: Formulations 5, 8, and 9 showed extreme viscosity and could not be injected, so the discharge rate was not determined.

**Table 4:** Burst release percentages of progesterone gels within 8 h.

Formulation	Burst Release Percentage (AVG ± SD, %)		
	2 h	4 h	8 h
15% Polymer	12.58 ± 2.96	20.37 ± 2.99	29.51 ± 3.77
20% Polymer	12.59 ± 2.48	20.57 ± 1.73	29.8 ± 1.69
30% Polymer	6.95 ± 0.64	13.12 ± 1.86	18.61 ± 1.60
35% Polymer	6.14 ± 0.21	11.61 ± 0.06	16.74 ± 0.47

**Table 5:** Semi-quantitative histological scores of subcutaneous tissues.

No.	Groups	Histological Score *
1	Control group	0
2	20% Polymer gel group	1
3	30% Polymer gel group	1
4	35% Polymer gel group	1

* Histological score represents the semi-quantitative histological evaluation index.

## Data Availability

The original contributions presented in this study are included in the article. Further inquiries can be directed to the corresponding author.
